# All-Cause Mortality and Its Predictors in Haemato-Oncology Patients with Febrile Neutropenia

**DOI:** 10.3390/jcm12175635

**Published:** 2023-08-29

**Authors:** Haim Shmuely, Lea Monely, Lev Shvidel

**Affiliations:** 1 Department of Internal Medicine D, Kaplan Medical Center, Rehovot 7612001, Israel; leamo@clalit.org.il; 2 Faculty of Medicine, Hebrew University, Jerusalem 9112001, Israel; levsh@clalit.org.il; 3 Institute of Hematology, Kaplan Medical Center, Rehovot 7612001, Israel

**Keywords:** febrile neutropenia, haemato-oncological patients, predictors of mortality, all-cause mortality

## Abstract

Febrile neutropenia (FN) is one of the most important life-threatening complications in haemato-oncology. Our objective was to report all-cause mortality rates in patients ill with a hematological malignancy (HM) hospitalized with a first FN episode and to identify predictors for mortality. We conducted a historical retrospective cohort study of consecutive patients with an HM, >18 years of age, admitted between January 2012 and August 2018 for a first episode of FN. Data on all-cause mortality 12 months after admission for FN were obtained. The Kaplan–Meier curve was used to describe mortality during the follow-up period. Univariate and multivariable analyses identified predictors for 1,3 and 12-month mortality. One hundred and fifty-eight patients (mean age 69.5, 49.4% males) were included. Overall, 54 patients died (15.8%, 25.9%, and 34.1% died after 1, 3, and 12 months, respectively). Lower serum albumin, higher serum gamma-glutamyl transferase (GGT), lower estimated glomerular filtration rate (eGFR), older age, higher temperature, and lower absolute lymphocyte count at admission were independent predictors of all-cause mortality after 12 months. Further studies are needed to confirm our results and identify therapeutic strategies to improve survival.

## 1. Introduction

Febrile neutropenia (FN) is considered a medical emergency in patients ill with hematologic malignancies (HM) who have received chemotherapy treatment, generally requiring immediate hospitalization and administration of empiric broad-spectrum antibiotics [1,2]. FN is associated with significant morbidity and mortality (~10% to 30%) among patients hospitalized for FN-related complications. Higher rates have been observed in patients with major comorbidities [1,3,4,5]. Risk factors for short-term mortality due to FN include age, cancer type, comorbidities, delayed administration of antibiotics, and laboratory abnormalities (i.e., low serum albumin, anemia, and increased lactate dehydrogenase) [6,7,8,9]. Nevertheless, there are limited data on longer-term outcomes post-FN and their associated risk factors. Lyman et al. found only an increased risk of mortality in patients requiring repeated hospitalizations for FN [10]. Another study described an increased long-term risk of infections in surviving patients afflicted with FN during chemotherapy [11]. FN might have a significant impact on morbidity and an indirect effect on mortality due to delays in chemotherapy administration and dosing alterations [12].

Our objective was to report on the short and long-term all-cause mortality rates in patients ill with HM admitted to our institution due to a first episode of FN and to identify predictors for mortality at the 1, 3, and 12-month follow-up.

## 2. Materials and Methods

### 2.1. Design, Participants, and Setting

We performed a retrospective historical cohort study of adult patients (>18 years) diagnosed with HM and hospitalized for FN at the Haemato-Oncology Unit, Kaplan Medical Center, Israel, between January 2012 and August 2018. Kaplan Medical Center is located in central Israel and is affiliated with the Hebrew University, Jerusalem, Israel. The Haemato-Oncology Unit is an acute care unit with five single, high-efficiency particulate air-filtered positive air pressure rooms. Patients were included in the study after experiencing their first episode of FN during the study period. FN is defined as a fever episode ≥38.3 °C or ≥38 °C lasting for one hour with an absolute neutrophil count (ANC) < 500/mm [13]. The study design was approved by the Institutional Review Board of the hospital (IRB #0204-17). There was no need for informed consent as this was a retrospective study.

### 2.2. Variables and Data Source

The following information was collected after reviewing the patient’s medical charts on admission: age, gender, body mass index, comorbidity defined as the presence and diagnosis of heart failure, diabetes mellitus, chronic pulmonary disease, chronic liver disease, and chronic renal failure. Clinical manifestations, the presumed source of infection, empirical antimicrobial therapy given during the first 48 h prior to receiving the susceptibility test results, and the presence of a central venous catheter were reported.

Laboratory results recorded were white blood cell, ANC, lymphocyte, and thrombocyte count. Profound neutropenia was defined as an ANC < 100/mm^3^. Calculated estimated glomerular filtration rate (eGFR) using the modification of diet in the renal disease (MDRD) method [13], C-reactive protein, serum glucose, bilirubin, lactic dehydrogenase; alkaline phosphatase, gamma-glutamyl transferase (GGT), glutamate oxaloacetate transaminase (GOT) and glutamic pyruvic transaminase levels, were also recorded.

The susceptibility of bacterial isolates from blood cultures was tested by disk diffusion and defined according to the Clinical and Laboratory Standards criteria [14]. The appropriateness of the empirical therapy was defined as matching the in vitro susceptibility of subsequently isolated bacteria from blood cultures. Data on all-cause mortality after admission for FN were obtained from the Israeli Ministry of Health’s database. The last date of follow-up was 31 August 2019. Patients who were alive until this date were censored. One-month, 3- and 12-month all-cause mortality was defined as the study outcome.

### 2.3. Statistical Analysis

Categorical variables were summarized as frequency and percentage. Continuous variables were evaluated for normal distribution using a histogram and Q-Q plot. Normally distributed continuous variables were reported as mean and standard deviation; skewed data were reported as median and interquartile range. The chi-square and Fisher exact test examined the association between categorical predictors and the 1- and 3-month all-cause mortality. An independent sample T and Mann-Whitney U test compared the continuous variables. A multivariable logistic regression examined the independent association with 1-month and 3-month all-cause mortality. The Kaplan–Meier curve and log-rank test examined the association between the categorical variables and 12-month all-cause mortality.

The univariate Cox regression examined the association between the 12-month mortality and continuous variables. The multivariable Cox regression examined the independent association with 12-month all-cause mortality. Variables found to be significantly associated with the study outcome were included in the multivariable analysis. The backward method was applied using *p* > 0.1 in the Wald test as criteria for variable removal. All statistical tests were two-sided; *p* < 0.05 was considered significant. In order to control for multiple comparisons, *p*-values were also adjusted using the false discovery rate (FDR) method. SPSS software statistically analyzed the data (IBM SPSS statistics for Windows, version 27, IBM cooperation, Armonk, NY, USA).

## 3. Results

### 3.1. Study Population

During the study period, 158 HM patients were admitted for FN. Median age was 69.5 (IQR, 58–76); 49.3% were male. The most frequent hematological malignancy found was lymphoma in 80 (50.6%) patients (Table S1), followed by acute myeloid leukemia (AML) in 37 (23.4%), myelodysplastic syndrome (MDS) in 17 (10.8%), multiple myeloma (MM) in 15 (9.5%), chronic lymphocytic leukemia (CLL) in 7 (4.4%) and in others, 2 (1.3%). The majority of lymphoma, AML, MDS, and CLL patients received first-line therapy prior to FN, except for the MM patients (Table S2). Diabetes mellitus was the most frequent comorbidity (21.5%). Antibiotic prophylaxis was prescribed to 46% of all FN patients, and anti-fungal prophylaxis was prescribed to all AML and transplanted patients. The demographic data and underlying diseases of the patients are shown in Table 1.

### 3.2. All-Cause Mortality during the Follow-Up Period

**Figure 1:**
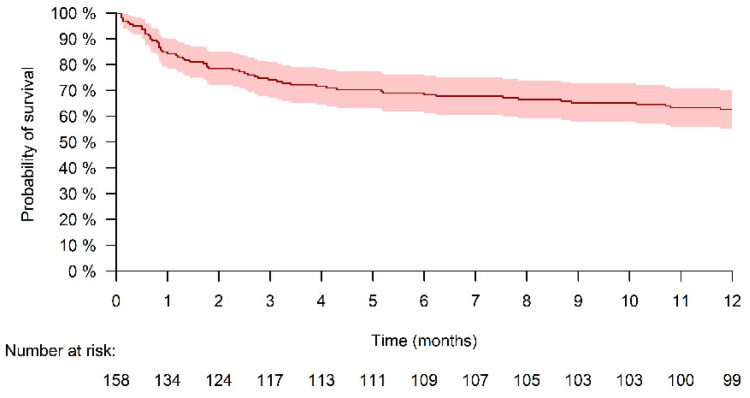
Kaplan–Meier curve demonstrating the all-cause mortality rate during the 12-month post-admission period. The colored shadow areas indicate a 95% confidence interval.

During the 12-month follow-up period, 54 patients died; 15.8% died after the first month, 25.9% after 3 months, and 34.1% after 12 months (Figure 1).

### 3.3. Predictors for All-Cause Mortality after 1, 3, and 12 Months

On univariate analysis, older age, higher temperature, lower serum albumin, lower eGFR, higher serum GGT, and a higher total and direct bilirubin on admission for FN were found to be significantly associated with all-cause mortality at all-time points (Table 2 and Table 3).

Higher serum lactic dehydrogenase and GOT levels were significantly associated with all-cause mortality after 1 and 3 months from FN admission (Table 2 and Table S3). Higher serum alkaline phosphatase levels on admission were associated with a 3-month mortality (Table S3). Lower total serum protein on admission was significantly associated with all-cause mortality after a 3- and 12-month follow-up (Table 3 and Table S3). Type of HM and lower absolute lymphocyte platelet count were significantly associated with all-cause mortality after 12 months (Table 3). On multivariate analysis (Table 4), lower serum albumin and higher serum GGT were found to be independent predictors of mortality at all-time points. Older age, lower eGFR, and higher temperature were independent predictors of mortality after 3 and 12 months. Lower absolute lymphocyte count was an independent predictor of mortality after 12 months.

### 3.4. Site of Infection, Bloodstream Infections and Antibiotic Treatment

The site of infection was diagnosed as FN of unknown origin in 106 (67%) of FN episodes. The most common recorded infection was a bloodstream infection (13%), followed by pneumonia (6%), a gastrointestinal infection (4%), soft tissue (3%), central line, urinary tract (3%) and other (2%). Blood cultures revealed pathogens in 21 cases: 15 (71%) Gram-negative bacteremia: *Escherichia coli* extended-spectrum beta-lactamase (ESBL) positive (3), *E. coli* ESBL negative (3), *Pseudomonas aeruginosa* (3), *Bacteroide’s fragilis* (2)*, Klebsiella* ESBL negative, *Salmonella group D*, *Carbapenem-resistant Enterobacteriaceae,* and *Campylobacter jejunii* (each one). Gram-positive bacteremia was cultured from 6 patients (29%): *Coagulase-negative staphylococcus* (2), *Methicillin sensitive staphylococcus* (2), and *Enterococcus faecalis* (2). Piperacillin-tazobactam therapy was initiated in 111 episodes; ceftazidime therapy was recorded in 45 episodes. Carbapenem therapy was administered to 10 patients. A glycopeptide antibiotic was added to the initial treatment in 21 episodes. Patients received appropriate empirical antibiotic treatment in 18/21 (86%) of bloodstream infections. Overall, appropriate empirical antibiotic treatment was not found to be significantly associated with 1-, 3- and 12-month mortality.

## 4. Discussion

Patients undergoing therapy for HM are at a significant risk of a life-threatening infection due to the immune-suppressive nature of their underlying disease, as well as chemotherapy-induced neutropenia [15]. FN is an infectious critical complication with a high mortality rate. Hence, broad-spectrum antimicrobial therapy should be initiated as swiftly as possible [16,17,18].

In this retrospective cohort study, we summarized the data of 158 consecutive HM patients hospitalized with a first episode of FN in a university-affiliated medical center. Fifty-four patients died during the 12-month follow-up period: 15.8% within the first month, 25.9% after three months, and 34.1% within the first year after their FN episode. The mortality rate of FN has been reported as 10–30% [1,3,4,5]. Moreover, FN frequently leads to chemotherapy dose reductions and treatment delays, thus compromising long-term clinical outcomes in responsive and potentially curable malignancies [19,20]. We included patients with HMs associated with a low survival rate, i.e., acute myeloid leukemia (23.4%) and myelodysplastic syndrome (10.8%). After a 12-month follow-up, using univariate analysis, the type of primary HM was significantly associated with a higher mortality rate (Table 3). However, multivariate analysis (Table 4) found that the type of primary HM was not independently associated with all-cause mortality at any point, probably due to a multifactorial effect. Furthermore, we observed that lower serum albumin and higher serum GGT levels on admission were independent predictors associated with all-cause mortality 1, 3, and 12 months after admission.

Serum albumin, a commonly used marker, examines the nutritional status of cancer patients. Malnutrition and inflammation suppress albumin synthesis, and a low serum albumin level can reflect disease severity, shown to be a strong predictor of prognosis [21,22]. Abnormal liver enzymes have been reported as independent risk factors for 30-day mortality after FN [23]. Gamma-glutamyl transferase (GGT), a key enzyme in the metabolism of glutathione, performs important roles in antioxidant mechanisms. Higher serum GGT was found to be an independent predictor of mortality at all-time points and has also been known as a predictor of mortality in the general population. GGT level, even within the reference range, was associated with an increased all-cause and cardiovascular mortality after adjusting for confounding factors [24,25,26]. Recently, GGT was reported as an independent predictor of overall and disease-specific mortality, with a dose–response relationship in a general population [27]. The underlying mechanism and clinical implications of GGT-based prognostication warrant further investigation.

Older age (adjusted odds ratio (OR) 1.037, *p* = 0.077, adjusted hazard ratio (HR) 1.032, *p* = 0.002), higher temperature on admission (adjusted OR 3.20, *p* = 0.01; and adjusted HR 1.601, *p* = 0.033) were independent predictors associated with all-cause mortality after 3 and 12 months, respectively. In previous studies of adults with FN, older age emerged as a significant risk factor for poor outcomes [28,29]. As for a higher temperature detected on admission as a predictor of mortality, trends towards higher mortality in neutropenic sepsis have been observed at >39.5 to 40 °C in British, Japanese, and Korean ICU sepsis cohorts [30,31], raising the possibility that there is a temperature threshold above which fever becomes a detriment in neutropenic sepsis, but not in non-neutropenic sepsis. Alternatively, it is possible that certain patients with neutropenic sepsis exhibited very high fevers due to an underlying malignancy rather than an infection. In our study, decreased eGFR was found independently associated with increased risk of 3 and 12-month all-cause mortality (Table 3 and Table S3).

In a previous study, Iff et al. stated that low eGFR was a risk factor for cancer deaths and a predictor of poor cancer outcomes in an older population [32]. The observed increased risk of mortality in patients with reduced eGFR is considerable. Thus, we suggest that decreased kidney function itself is a predictor of poor outcomes. There are a few potential reasons for this suggestion. Firstly, previous studies have shown that patients with chronic kidney diseases are less likely to undergo cancer screening and are afflicted with multiple comorbid conditions [33]. Secondly, cancer management in patients with decreased kidney function is complex. There is scant information as to the optimal timing and necessary dose adjustment of cytotoxic agents for patients with reduced kidney function.

Lower absolute lymphocyte count was found to be an independent predictor of mortality after 12 months. Blay et al. [34] reported that early lymphopenia occurring after cytotoxic chemotherapy was a risk factor for FN and could be a sensitivity marker to the hematologic toxicity of chemotherapeutic agents since chemotherapy induces lymphopenia before it induces neutropenia. Another possible interpretation is that lymphocytes may play a role in the restoration of normal hematopoiesis after cytotoxic chemotherapy. The decrease in lymphocyte counts results in the reduced production of cytokines, thereby interfering with the restoration of normal neutrophil counts [35].

Generally, fever is the only sign of an infection, with 60% of febrile episodes presenting in neutropenic patients. This condition, defined as FN of an unknown origin, was detected in 67% of our patients. Because 70% of these patients responded to empirical antimicrobial treatment, it is most likely that many of these episodes represented undetected infections, which, if left untreated, share a considerable mortality, especially if they represent an occult Gram-negative bacteremia [36]. Bloodstream infection rates, reported between 11% and 38% in other studies, were 26% in our study [37,38]. Lo Menzo et al. reported that the most common infections in FN patients were in the respiratory tract, followed by the bloodstream and urinary tract [39]. In our study, bloodstream infections were the most common, followed by pneumonia and gastrointestinal infections. The rates of Gram-negative bacteria have increased in recent years in FN patients [40,41]. A mucosal injury of the gut due to chemotherapy causes Gram-negative bacteremia to emerge from the intestinal flora [34]. Gedik et al. [42] reported that most of the isolated Gram-negative bacteria did not produce ESBL in the infections of FN patients with HM. In our study, Gram-negative ESBL-producing and ESBL-negative spp. were equally cultured from the blood. *Escherichia coli* was prominent in the bloodstream infections.

Early and appropriate antimicrobial therapy with broad-spectrum antibiotics appears to reduce mortality [43,44]. An antipseudomonal beta lactam-beta lactamase inhibitor antibiotic, also active against ESBL-positive Enterobacteriaceae spp., can be the first choice for empirical therapy of FN. If the patient has a history of resistant bacterial infection or colonization or a history of hospitalization in the ICU, the spectrum of antimicrobials may be broader [45]. In our cohort, piperacillin-tazobactam and ceftazidime carbapenem therapy were administrated with a glycopeptide antibiotic added to the initial treatment in line with the de-escalation strategy. Overall, appropriate empirical antibiotic treatment, 18/21 (86%), was not significantly associated with 1-, 3-, and 12-month mortality, probably due to the limited number of positive blood cultures in this cohort.

Our study has several limitations. This study took place in a single site, a university-hospital, that might not be generalizable to other settings. Secondly, some data are missing due to the retrospective study design. None of the FN patients in our cohort had a MASCC score documented on admission. Data on all-cause mortality after admission for FN were obtained from the Israeli Ministry of the Interior’s database, which does not provide the cause of death. Since the number of positive blood cultures was low, it was difficult to assess the correlation between the appropriateness of treatment and mortality. The cited references on FN include patients with solid cancers, not primarily hematological patients. Therefore, a comparison of the data is limited as these patient cohorts are often different in therapy and clinical course. However, the study results are consistent with previous studies, which increases the external validity of the study. Major strengths of the study include the long-term follow-up and access to precise and complete nationwide data on all-cause mortality obtained through electronic health records and the Israeli Ministry of the Interior’s database.

## 5. Conclusions

We found that higher serum GGT levels, older age, higher peak temperature, and low absolute lymphocyte counts on admission of FN patients are important predictors of 12-month mortality after a first episode of FN in HM patients. Further prospective studies are warranted to confirm our results and identify various therapeutic strategies to improve survival.

## Figures and Tables

**Table 1:** Characteristics of 158 patients admitted for febrile neutropenia and site of infection.

Characteristics	
Sex (male) *n* (%)	78 (49.4%)
Age median (IQR)	69.54 (58.43–76.23)
BMI median (IQR)	24.8 (22–29.25)
Temperature (°C) median (IQR)	38.5 (38.1–39)
Line	15 (9.5%)
HM *n* (%)	
Lymphoma	80 (50.6%)
Acute myeloid leukemia	37 (23.4%)
Myelodysplastic syndrome	17 (10.8%)
Multiple myeloma	15 (9.5%)
Chronic lymphocytic leukemia	7 (4.4%)
Other	2 (1.3%)
Comorbidities *n* (%)	
DM	34 (21.5%)
CRF	31 (19.6%)
IHD	17 (10.8%)
CHF	13 (8.2%)
Laboratory at admission for FN median (IQR)	
Hemoglobin (12–16 g/dL)	9.1 (8–10.2)
WBC (4.5–11 K/µL)	1.4 (0.6–3.3)
Absolute neutrophils (1–4.8 K/µL)	0.23 (0.1–0.44)
ALC (1.0–4.8 K/µL)	0.45 (0.2–0.99)
Platelets (150–450 K/µL)	81 (27–139.75)
CRP (0–0.5 mg/dL)	86.7 (67.4–254)
ESR (2–30 mm/h)	52 (32–96)
eGFR (mL/min/1.73 m^2^)	88.22 (67.24–112.16)
Protein (5.8–8.3 g/dL)	5.95 (0.98)
Albumin (3.2–4.6 g/dL)	3.29 (0.55)
Bilirubin total (0.3–1.2 mg/dL)	0.7 (0.5–1)
Alkaline phosphatase (40–150 U/L)	77 (60.5–101.25)
GGT (9–36 U/L)	40 (20.25–93.75)
GOT (5–34 U/L)	19 (14–27)
GPT (0–55 U/L)	17.5 (12–31)

Laboratory at the onset of febrile neutropenia is indicated in the median interquartile range. Percentages are indicated in parentheses. *HM*—hematologic malignancies; *FA*—febrile neutropenia; *ICQ*—interquartile range; *DM*—diabetes mellitus; *CRF*—chronic renal failure; *IHD*—ischemic heart disease; *CHF*—congestive heart failure; *WBC*—white blood cells; *ALC*—absolute lymphocyte count; *CRP*—C-reactive protein; *ESR*—erythrocyte sedimentation rate; *eGFR*—estimating glomerular filtration rate; *GGT*—gamma-glutamyl transferase; *GOT*—glutamate oxaloacetate transaminase; *GPT*—glutamic pyruvic transaminase.

**Table 2:** Univariate analysis of continuous and categorical predictors for 1-month mortality.

Predictor		Mortality			
		No (*n* = 133)	Yes (*n* = 25)	*p*	Adj. *p*
Age (years)	median (IQR)	68.4 (55.2–74.5)	76.2 (67.7–80.8)	0.003	0.017
Male	*n* (%)	67 (50.4%)	11 (44%)	0.559	0.648
BMI (Kg/m^2^)	median (IQR)	24.6 (21.8–29.1)	28 (23.8–32.6)	0.198	0.359
Temperature (°C)	median (IQR)	38.4 (38.1–38.8)	38.8 (38.2–39.1)	0.045	0.131
HM					
Lymphoma	*n* (%)	66 (49.6%)	14 (56%)	0.723	0.806
CLL	*n* (%)	7 (5.3%)	0 (0%)		
AML	*n* (%)	34 (25.6%)	5 (20%)		
MM	*n* (%)	13 (9.8%)	2 (8%)		
MDS	*n* (%)	13 (9.8%)	4 (16%)		
Comorbidities					
DM	*n* (%)	27 (20.3%)	7 (28%)	0.390	0.566
CRF	*n* (%)	22 (16.5%)	9 (36%)	0.050	0.132
CLD	*n* (%)	20 (15%)	4 (16%)	>0.999	>0.999
IHD	*n* (%)	13 (9.8%)	4 (16%)	0.478	0.630
CHF	*n* (%)	11 (8.3%)	2 (8%)	>0.999	>0.999
CCI	median (IQR)	5 (3–6)	6 (5–9)	0.006	0.029
Laboratory					
Hb (12–16 g/dL)	median (IQR)	9.2 (8.2–10.3)	8.6 (7.8–9.3)	0.082	0.183
WBC (4.5–11 K/µL)	median (IQR)	1.3 (0.6–3.1)	1.7 (0.8–4.6)	0.333	0.508
ANC (1–4.8 K/µL)	median (IQR)	0.2 (0.1–0.4)	0.3 (0.1–0.5)	0.273	0.440
ANC < 100	*n* (%)	29 (21.8%)	3 (12%)	0.415	0.573
ALC (1–4.8 K/µL)	median (IQR)	0.4 (0.2–0.9)	0.6 (0.2–1.2)	0.556	0.648
PLT (150–450 K/µL)	median (IQR)	85 (27–149.5)	54 (27–100.5)	0.240	0.409
CRP (0–0.5 mg/dL)	median (IQR)	86.5 (65.4–147.3)	156 (72.4–267)	0.198	0.359
ESR (2–30/mmh)	median (IQR)	46 (31.3–85.8)	129 (79–0)	0.060	0.145
eGFR (mL/min/1.73 m^2^)	median (IQR	91.8 (71.5–116.5)	73.8 (44.1–91.8)	0.003	0.017
Protein (6.4–6.3 g/dL)	median (IQR)	6.02 (0.9)	5.6 (1.27)	0.125	0.259
Albumin (3.2–4.6 g/dL)	median (IQR)	3.39 (0.5)	2.74 (0.47)	<0.001	0.015
LDH (125–220 U/L)	median (IQR)	401 (280–564.5)	612 (325–854)	0.022	0.080
ALP (40–150 U/L)	median (IQR)	76 (61–102.5)	85 (55.5–101)	0.922	0.990
GGT (9–36 U/L)	median (IQR)	36 (20–76)	93 (28.5–172.5)	0.003	0.017
GOT (5–34 U/L)	median (IQR)	18 (13–25.5)	23 (16.5–34.5)	0.029	0.093
GPT (0–55 U/L)	median (IQR)	17 (12–29.5)	21 (12.5–34.5)	0.528	0.648
Bilirubin total (0.3–1.2)	median (IQR)	0.7 (0.5–1)	0.9 (0.6–1.7)	0.020	0.080
Bilirubin direct (0.5 mg/dL)	median (IQR)	0.2 (0.1–0.4)	0.4 (0.2–0.8)	0.001	0.015

Values are presented as mean, standard deviation, or median interquartile range. IQR—interquartile range; BMI—body mass index; HM—hematological malignancies; CLL—chronic lymphatic leukemia; MM—multiple myeloma; AML—acute myelocytic leukemia; MDS—myelodysplastic syndrome; CHF—congestive heart failure; IHD—ischemic heart disease; CRF—chronic renal failure; CLD—chronic liver disease; Hb—hemoglobin; WBC—white blood cells; ANC—absolute neutrophil count; ALC—absolute lymphocyte; PLT—platelets; CRP—C-reactive protein; ESR—erythrocyte sedimentation rate; eGFR—estimated glomerular filtration rate; LDH—lactic dehydrogenase; ALP—alkaline phosphatase; GGT—gamma-glutamyl transferase; GOT—glutamate oxaloacetate transaminase; GPT—glutamic pyruvic transaminase; CCI—Charleson co-morbidity index.

**Table 3:** Univariate analysis of continuous and categorical predictors for 12-month mortality.

		Mortality			
Predictor		No (*n* = 104)	Yes (*n* = 54)	*p*	Adj. *p*
Age (years)	median (IQR)	64.81 (50.77–70.72)	75.4 (70.25–81.22)	<0.001	0.002
Male	*n* (%)	50 (50.5%)	28 (47.5%)	0.735	0.799
BMI (Kg/m^2^)	median (IQR)	24.4 (21.35–29.55)	26.1 (22.75–29.05)	0.596	0.725
Temperature (°C)	median (IQR)	38.4 (38.1–38.7)	38.5 (38.18–39)	0.040	0.089
HM					
Lymphoma	*n* (%)	48 (48.5%)	32 (54.2%)	0.024	0.063
CLL	*n* (%)	7 (7.1%)	0 (0%)		
AML	*n* (%)	26 (26.3%)	13 (22%)		
MM	*n* (%)	12 (12.1%)	3 (5.1%)		
MDS	*n* (%)	6 (6.1%)	11 (18.6%)		
Comorbidities					
DM	*n* (%)	18 (18.2%)	16 (27.1%)	0.156	0.256
IHD	*n* (%)	11 (11.1%)	6 (10.2%)	0.932	0.932
CRF	*n* (%)	14 (14.1%)	17 (28.8%)	0.012	0.035
CLD	*n* (%)	14 (14.1%)	10 (16.9%)	0.658	0.734
CHF	*n* (%)	5 (5.1%)	8 (13.6%)	0.076	0.154
CCI	median (IQR)	5 (3–6)	6 (5–7)	0.002	0.008
Laboratory					
Hb (12–16 g/dL)	median (IQR)	9.2 (8.2–10.3)	8.6 (7.8–9.3)	0.082	0.154
WBC (4.5–11 K/µL)	median (IQR)	1.47 (0.64–3.77)	1.27 (0.4–2.57)	0.274	0.378
ANC (1–4.8 K/µL)	median (IQR)	0.2 (0.1–0.45)	0.25 (0.1–0.42)	0.625	0.725
ANC < 100	*n* (%)	26 (22.2%)	6 (14.6%)	0.370	0.488
ALC (1–4.8 K/µL)	median (IQR)	0.42 (0.2–1.1)	0.48 (0.2–0.9)	0.008	0.026
PLT (150–450 K/µL)	median (IQR)	102 (34–156)	43 (23–98)	0.004	0.015
CRP (0–0.5 mg/dL)	median (IQR)	87.35 (39.85–154)	83.8 (75.25–172.5)	0.108	0.184
ESR (2–30 mm/h)	median (IQR)	42 (31.5–75)	82 (30.75–130.75)	0.085	0.154
eGFR (mL/min/1.73 m^2^)	median (IQR	95.17 (75.51–119.24)	77.45 (49.86–94.12)	<0.001	0.002
Protein (6.4–6.3 g/dL)	median (IQR)	6.06 (0.85)	5.78 (1.14)	0.029	0.07
Albumin (3.2–4.6 g/dL)	median (IQR)	3.45 (0.49)	3.02 (0.55)	<0.001	0.002
LDH (125–220 U/L)	median (IQR)	405 (272–573)	446 (299–736)	0.260	0.378
ALP (40–150 U/L)	median (IQR)	72 (57–101)	83 (63–102)	0.271	0.378
GGT (9–36 U/L)	median (IQR)	36 (20–74.5)	63 (24–138)	<0.001	0.002
GOT (5–34 U/L)	median (IQR)	17 (13–24)	21 (14–29)	0.551	0.695
GPT (0–55 U/L)	median (IQR)	16 (12–29)	21 (12–34)	0.875	0.906
Bilirubin total (0.3–1.2)	median (IQR)	0.7 (0.5–1)	0.85 (0.6–1.28)	<0.001	0.002
Bilirubin direct (0.5 mg/dL)	median (IQR)	0.2 (0.1–0.33)	0.3 (0.2–0.6)	<0.001	0.002

Adj—adjusted; IQR—interquartile range; BMI—body mass index; HM—hematological malignancies; CLL—chronic lymphatic leukemia; MM—multiple myeloma; AML—acute myelocytic leukemia; MDS—myelodysplastic syndrome; CHF—congestive heart failure; IHD—ischemic heart disease; CRF—chronic renal failure; CLD—chronic liver disease; Hb—hemoglobin; WBC—white blood cells; ANC—absolute neutrophil count; ALC—absolute lymphocyte; PLT—platelets; CRP—C-reactive protein; ESR—erythrocyte sedimentation rate; eGFR—estimating glomerular filtration rate; LDH—lactic dehydrogenase; ALP—alkaline phosphatase; GGT—gamma-glutamyl transferase; GOT—glutamate oxaloacetate transaminase; GPT—glutamic pyruvic transaminase; CCI—Charleson co-morbidity index.

**Table 4:** Multivariate logistic and cox regression of predictor for mortality using the backward elimination method.

Mortality		Adj. OR (95% CI)	*p*
1 m	Albumin (g/dL)	0.096 (0.033–0.28)	<0.001
	GGT (10 IU/L)	1.073 (1.017–1.132)	0.010
3 m	Albumin(g/dL)	0.185 (0.071–0.481)	0.001
	GGT (10 IU/L)	1.079 (1.020–1.142)	0.009
	eGFR (mL/min/1.73 m^2^)	0.982 (0.966–0.999)	0.035
	Age (years)	1.037 (0.996–1.08)	0.077
	Temperature (°C)	3.209 (1.274–8.086)	0.013
Mortality		Adj. HR (95% CI)	*p*
12 m	Albumin(g/dL)	0.456 (0.27–0.771)	0.003
	GGT (10 IU/L)	1.033 (1.005–1.062)	0.020
	eGFR (mL/min/1.73 m^2^)	0.986 (0.977–0.995)	0.004
	Age (years)	1.032 (1.012–1.053)	0.002
	Temperature(°C)	1.601 (1.039–2.467)	0.033
	ALC (K/µL)	1.087 (1.025–1.152)	0.005

*GGT*—gamma-glutamyl transpeptidase; *eGFR*—estimated glomerular filtration rate; *ALC*—absolute lymphocyte count.

## Data Availability

The datasets generated during and/or analyzed during the current study are available from the corresponding author upon reasonable request.

## Supplementary Materials

The following supporting information can be downloaded at: https://www.mdpi.com/article/10.3390/jcm12175635/s1. Table S1: List of lymphoma patients with neutropenic fever. Table S2: Last line of therapy prior to febrile neutropenic episode. First line treatment and salvage treatment. Table S3: Univariate analysis of continuous and categorical predictors for 3-month mortality.
