# Supplementary material for: All-Cause Mortality and Its Predictors in Haemato-Oncology Patients with Febrile Neutropenia
Source: J Clin Med. 2023 Aug 29;12(17):5635. doi: 10.3390/jcm12175635 (PMC10489066; doi:10.3390/jcm12175635)
Supplement: Supplementary file 1 [file jcm-12-05635-s001.zip › jcm-12-05635-s001.pdf]

Table S1: List of lymphoma patients with neutropenic fever

Characteristics		Patients (n)
Indolent B cell Lymphoma		15
Follicular	(6)	
Marginal zone	(6)	
Small cell	(3)	
Aggressive		59
DLBC	(51)	
Mantle cell	(6)	
Burkiitt	(2)	
Peripheral T-cell		2
Hodgkin		4

DLBC: Diffuse large B-cell lymphoma

Table S2. Last line of therapy prior to febrile neutropenic episode. First line treatment and salvage treatment

Hematological malignancy	Treatment	Patients (N)
Lymphoma (80)	First line	60
	Salvage	20
AML (37)	First line	30
	Salvage	7
MDS (17)	First line	13
	Salvage	4
MM (15)	First line	6
	Salvage	9
CLL (7)	First line	7

Lymphoma: examples for first line: CHOP-R (Cyclophosphamide-Hydroxydoxydoxorubcin-Oncovin-Prednisone-Rituximab); Bendamustine-Obinutuzumab; Bendamustine -Rituximab
Salvage: ICE; Gemcytabine-Oxaliplatine
AML: examples for first line: 3+7; Arabinoside-Idarubicin; Venetoclax-Azatidine; high dose Cytarabine
Salvage: high dose Arabinoside-Mitoxantrone
Multiple Myeloma: examples for first line: Bortezomib- Lenalidomide-Dexamethasone; Bortezomib-Cyclophosphamide -Dexamethasone; High dose Melphalan with autologous stem cell salvage
CLL: examples for first line: Fludarabine -Cyclophosphamide-Rituximab; Bendamustine-Rituximab

Table S3 Univariate analysis of continuous and categorical predictors for 3-month mortality*

		Mortality			
**Predictor**		**No (n=117)**	**Yes (n=41)**	**P**	**Adj. p**
Age (years)	median (IQR)	67.1 (52.2-73.1)	76.2 (70.5-82)	<0.001	0.003
Male n (%)	n (%)	57 (48.7%)	21 (51.2%)	0.783	0.841
BMI (kg/m^2)	median (IQR)	24.5 (21.9-29.6)	25 (22.3-29.1)	0.692	0.807
Temperature (C°)	median (IQR	38.4 (38-38.7)	38.8 (38.3-39.1)	0.001	0.004
HM					
Lymphoma	n (%)	57 (48.7%)	23 (56.1%)	0.207	0.334
CLL	n (%)	7 (6%)	0 (0%)		
AML	n (%)	30 (25.6%)	9 (22%)		
MM	n (%)	13 (11.1%)	2 (4.9%)		
MDS	n (%)	10 (8.5%)	7 (17.1%)		
Comorbidities					
DM	n (%)	23 (19.7%)	11 (26.8%)	0.336	0.513
IHD	n (%)	12 (10.3%)	5 (12.2%)	0.772	0.841
CRF	n (%)	19 (16.2%)	12 (29.3%)	0.071	0.137
CLD	n (%)	17 (14.5%)	7 (17.1%)	0.696	0.807
CHF	n (%)	7 (6%)	6 (14.6%)	0.101	0.183
CCI	median (IQR)	5 (3-6)	6 (5-7)	0.004	0.013
Laboratory results					
Hb (12-16 g/dl)	median (IQR)	9.3 (8.1-10.5)	8.9 (7.9-9.3)	0.051	0.106
WBC (4.5-11 K/µl)	median (IQR)	1.4 (0.6-3.1)	1.3 (0.6-3.7)	0.812	0.841
ANC (1-4.8 K/µl)	median (IQR)	0.2 (0.1-0.4)	0.3 (0.1-0.5)	0.442	0.61
ANC <100	n (%))	26 (22.2%)	6 (14.6%)	0.370	0.536
ALC (1-4.8 K/µl)	median (IQR)	0.4 (0.2-0.9)	0.6 (0.2-1.2)	0.561	0.106
PLT (150-450 K/µl)	median (IQR)	89 (27-151.5)	49 (25-100)	0.041	0.841
CRP (0-0.5 mg/dl)	median (IQR)	86.3 (59.4-145)	116 (77.2-209.3)	0.183	0.61
ESR (0-0.5 mg/dl)	median (IQR)	50 (32-96)	67.5 (18.3-118)	0.925	0.536
eGFR (mL/min/1.73m^2)	median (IQR	93.4 (73.6-118.8)	75.1 (38.3-99.3)	<0.001	0.707
Protein (6.4-6.3 g/dl)	median (IQR)	6.1 (0.88)	5.54 (1.12)	0.001	0.092

Albumin (3.2-4.6 g/dl)	median (IQR)	3.44 (0.49)	2.85 (0.49)	<0.001	0.312
LDH (125-220 U/L)	median (IQR)	400 (276.5-536.5)	532 (347.5-854)	0.007	0.925
ALP (40-150 U/L)	median (IQR)	72 (57.5-92.5)	89 (64.5-135)	0.025	0.003
GGT (9-36 U/L)	median (IQR)	35 (20-66)	93 (31-158.5)	<0.001	0.004
GOT (5-34 U/L)	median (IQR)	17 (13-24)	23 (18.5-32.5)	0.002	0.003
GPT (0-55 U/L)	median (IQR)	16 (12-29.5)	21 (12-34.5)	0.544	0.02
Bilirubin total (0.3-1.2)	median (IQR)	0.7 (0.5-1)	0.9 (0.6-1.6)	0.014	0.06
Bilirubin direct (0.5mg/dl)	median (IQR)	0.2 (0.1-0.4)	0.4 (0.2-0.7)	<0.001	0.003

Values are presented as mean standard deviation or median interquartile range.

IQR: interquartile range; SD: standard deviation; BMI: body mass index; HM: hematological malignancies; CLL: chronic lymphatic leukemia; MM: multiple myeloma; AML: acute myelocytic leukemia; MDS: myelodysplastic syndrome; CHF: congestive heart failure; IHD: ischemic heart disease; CRF: chronic renal failure; CLD: chronic liver disease; GI: gastrointestinal disease; Hb: hemoglobin; WBC: white blood cells; ANC: absolute neutrophil count; ALC: absolute lymphocyte; PLT: platelets; CRP: C-reactive protein; ESR: erythrocyte sedimentation rate; eGFR: estimating glomerular filtration rate; LDH: lactic dehydrogenase; ALP: alkaline phosphatase; GGT: gamma glutamyl transferase; GOT: glutamate oxaloacetate transaminase; GPT: glutamic pyruvic transaminase.
